# Supplementary material for: Is it feasible to detect FLOSS version release events from textual messages? A case study on Stack Overflow
Source: PLoS One. 2021 Feb 4;16(2):e0246464. doi: 10.1371/journal.pone.0246464 (PMC7861391; doi:10.1371/journal.pone.0246464)
Supplement: S1 Appendix — (PDF) [file pone.0246464.s001.pdf]

S1 Appendix Goodness of fit measures of Logistic Regression models. In the current appendix we provide detailed information on the goodness of fit for the Logistic Regression models (Tables S1 and S2).Tjur R^2 and Adjusted McFadden (Adj. MF) R^2 are used to choose the best fitted model whose detailed analysis is described in the paper. Event-based multiple packages datasets were not considered due to violation of the independence condition of the Logistic Regression model. Based on the goodness of fit, we have chosen the Selenium package, minor updates, event-based time steps dataset. Not all the statistical tools are meant to be used with the numbers of features considered in the Table S2 - Tjur R^2 does not fully adjust to the larger numbers of features, leading to erroneous results. Adjusted McFadden R^2 is a more appropriate measure for the hSBM feature space interpretation.

Table S1. Logistic Regression models, LDA: goodness of fit

	AIC	LLR Test	Tjur R^2	Adj. McFadden R^2	Number of Features
Multiple major event-based	117.27	0.17	0.20	-0.16	18
Multiple minor event-based	306.04	<0.001*	0.40	0.27	10
Multiple patch event-based	423.54	<0.001*	0.23	0.18	1
Django minor event-based	311.03	<0.001*	0.07	0.05	4
Django patch event-based	130.02	0.10	0.02	-0.03	2
Selenium minor event-based	320.96	<0.001*	0.16	0.07	12
Selenium patch event-based	135.06	0.17	0.00	-0.03	1
Multiple major c.w.-based	165.83	0.62	0.06	-0.17	18
Multiple minor c.w.-based	457.04	<0.001*	0.13	0.04	15
Multiple patch c.w.-based	480.45	<0.001*	0.13	0.03	17
Django minor c.w.-based	200.83	0.01	0.05	0.01	4
Django patch c.w.-based	95.57	<0.001*	0.13	0.05	5
Selenium minor c.w.-based	306.02	<0.001*	0.10	0.05	8
Selenium patch c.w.-based	151.27	0.36	0.04	-0.12	14

The table provides a list of goodness of fit measures for Logistic Regression models, all datasets, LDA feature space. "c.w.-based" corresponds to the calendar week-based time step datasets. The Number of Features column corresponds to the number of features in the model after the RFECV feature selection step. Significant model fits based on Log-likelihood Ratio test are marked with a star(*).

Table S2. Logistic Regression models, hSBM: goodness of fit

	AIC	LLR Test	Tjur R^2	Adj. McFadden R^2	Number of Features
Multiple major event-based	246.00	0.90	1.00	-1.41	675
Multiple minor event-based	642.09	0.004	0.99	-0.52	407
Multiple patch event-based	484.17	<0.001*	0.09	0.06	2
Django minor event-based	270.00	<0.001*	1.00	0.21	139
Django patch event-based	636.00	1.00	1.00	-3.81	608
Selenium minor event-based	345.82	0.05	0.01	-0.01	2
Selenium patch event-based	131.00	0.01	0.02	-0.00	2
Multiple major c.w.-based	153.46	0.02	0.05	-0.00	5
Multiple minor c.w.-based	294.09	<0.001*	0.72	0.39	72
Multiple patch c.w.-based	500.97	0.01	0.03	0.00	5
Django minor c.w.-based	728.00	1.00	1.00	-2.44	474
Django patch c.w.-based	85.25	<0.001*	0.20	0.22	5
Selenium minor c.w.-based	248.35	<0.001*	0.68	0.23	72
Selenium patch c.w.-based	710.00	1.00	1.00	-4.00	407

The table provides a list of goodness of fit measures for Logistic Regression models, all datasets, hSBM feature space. "c.w.-based" corresponds to the calendar week-based time step datasets. The Number of Features column corresponds to the number of features in the model after RFECV feature selection step. Significant model fits based on Log-likelihood Ratio test are marked with a star(*).
