# Supplementary material for: Is it feasible to detect FLOSS version release events from textual messages? A case study on Stack Overflow
Source: PLoS One. 2021 Feb 4;16(2):e0246464. doi: 10.1371/journal.pone.0246464 (PMC7861391; doi:10.1371/journal.pone.0246464)
Supplement: S2 Appendix — (PDF) [file pone.0246464.s002.pdf]

S2 Appendix Detailed performance of estimators.

The appendix provides detailed results of the study using various performance metrics and the permutation test (Tables S1,S2). Due to the class imbalance we report mean values over two cases - events treated as a positive and a negative class. The exception is the ROC-AUC metric which we compute with events as a positive class - its mean is always 0.5 by definition.

Table S1. Model performance, LDA feature space

	Number of features	ROC-AUC	PR-AUC	F1-score	P.test p-val
**CatBoost**					
Multiple major event-based	1	0.67	0.56	0.58	0.07
Multiple minor event-based	2	0.44	0.51	0.45	<0.001
Multiple patch event-based	1	0.59	0.51	0.46	<0.001
Django minor event-based	5	0.42	0.51	0.46	0.50
Django patch event-based	4	0.49	0.54	0.55	0.15
Selenium minor event-based	4	0.30	0.51	0.45	1.00
Selenium patch event-based	14	0.59	0.50	0.46	0.81
Multiple major c.w.-based	9	0.47	0.53	0.54	0.18
Multiple minor c.w.-based	2	0.57	0.51	0.37	<0.001
Multiple patch c.w.-based	2	0.51	0.50	0.52	0.40
Django minor c.w.-based	17	0.52	0.50	0.44	1.00
Django patch c.w.-based	2	0.44	0.50	0.53	0.21
Selenium minor c.w.-based	7	0.43	0.50	0.45	1.00
Selenium patch c.w.-based	2	0.54	0.51	0.46	1.00
**Random Forest**					
	Number of features	ROC-AUC	PR-AUC	F1-score	P.test p-val
Multiple major event-based	1	0.66	0.57	0.58	0.06
Multiple minor event-based	1	0.52	0.59	0.52	0.19
Multiple patch event-based	1	0.50	0.70	0.51	0.01
Django minor event-based	14	0.48	0.53	0.44	0.02
Django patch event-based	1	0.56	0.50	0.51	0.30
Selenium minor event-based	6	0.35	0.51	0.47	1.00
Selenium patch event-based	14	0.49	0.50	0.46	0.03
Multiple major c.w.-based	9	0.46	0.50	0.50	0.23
Multiple minor c.w.-based	7	0.60	0.51	0.50	0.22
Multiple patch c.w.-based	2	0.45	0.51	0.46	0.49
Django minor c.w.-based	10	0.57	0.50	0.44	1.00
Django patch c.w.-based	11	0.37	0.51	0.44	1.00
Selenium minor c.w.-based	18	0.31	0.51	0.48	1.00
Selenium patch c.w.-based	5	0.45	0.51	0.46	1.00
**Logistic Regression**					
	Number of features	ROC-AUC	PR-AUC	F1-score	P.test p-val
Multiple major event-based	18	0.32	0.40	0.49	0.88
Multiple minor event-based	10	0.38	0.45	0.45	0.93
Multiple patch event-based	1	0.52	0.50	0.46	0.98
Django minor event-based	4	0.53	0.51	0.43	0.36
Django patch event-based	2	0.37	0.47	0.47	0.44
Selenium minor event-based	12	0.37	0.46	0.47	0.98
Selenium patch event-based	1	0.54	0.52	0.45	0.46
Multiple major c.w.-based	18	0.42	0.48	0.48	0.80
Multiple minor c.w.-based	15	0.56	0.54	0.52	0.19
Multiple patch c.w.-based	17	0.46	0.46	0.32	0.89
Django minor c.w.-based	4	0.48	0.50	0.44	0.52
Django patch c.w.-based	5	0.42	0.48	0.47	0.86
Selenium minor c.w.-based	8	0.41	0.48	0.48	0.72
Selenium patch c.w.-based	14	0.60	0.52	0.47	0.21

In the table we report the number of features after the feature selection step together with ROC-AUC, PR-AUC, F1-score and permutation test p-value measures for LDA feature space.

Table S2. Model performance, hSBM feature space

	Number of features	ROC-AUC	PR-AUC	F1-score	P.test p-val
**CatBoost**					
Multiple major event-based	674	0.26	0.54	0.16	1.00
Multiple minor event-based	540	0.64	0.50	0.51	0.01
Multiple patch event-based	1	0.56	0.50	0.35	0.81
Django minor event-based	1	0.50	0.50	0.50	0.26
Django patch event-based	473	0.55	0.52	0.46	1.00
Selenium minor event-based	473	0.59	0.52	0.46	1.00
Selenium patch event-based	674	0.52	0.50	0.44	1.00
Multiple major c.w.-based	71	0.55	0.50	0.46	1.00
Multiple minor c.w.-based	4	0.47	0.50	0.48	0.03
Multiple patch c.w.-based	607	0.52	0.51	0.51	0.14
Django minor c.w.-based	138	0.47	0.51	0.46	0.04
Django patch c.w.-based	272	0.59	0.50	0.48	1.00
Selenium minor c.w.-based	205	0.62	0.51	0.47	1.00
Selenium patch c.w.-based	4	0.59	0.52	0.45	0.44
**Random Forest**					
	Number of features	ROC-AUC	PR-AUC	F1-score	P.test p-val
Multiple major event-based	138	0.71	0.52	0.16	1.00
Multiple minor event-based	1	0.50	0.75	0.49	<0.001
Multiple patch event-based	1	0.59	0.50	0.41	0.88
Django minor event-based	1	0.57	0.55	0.47	<0.001
Django patch event-based	1	0.64	0.54	0.47	1.00
Selenium minor event-based	607	0.48	0.50	0.46	1.00
Selenium patch event-based	1	0.43	0.51	0.44	1.00
Multiple major c.w.-based	138	0.48	0.50	0.46	1.00
Multiple minor c.w.-based	406	0.56	0.51	0.45	0.29
Multiple patch c.w.-based	473	0.45	0.51	0.40	<0.001
Django minor c.w.-based	1	0.52	0.51	0.45	1.00
Django patch c.w.-based	607	0.48	0.50	0.48	1.00
Selenium minor c.w.-based	607	0.60	0.56	0.35	0.07
Selenium patch c.w.-based	607	0.69	0.52	0.47	1.00
**Logistic Regression**					
	Number of features	ROC-AUC	PR-AUC	F1-score	P.test p-val
Multiple major event-based	674	0.29	0.52	0.20	0.76
Multiple minor event-based	406	0.60	0.50	0.36	0.90
Multiple patch event-based	1	0.37	0.51	0.49	<0.001
Django minor event-based	138	0.37	0.51	0.41	0.01
Django patch event-based	607	0.54	0.51	0.42	0.15
Selenium minor event-based	1	0.52	0.50	0.46	1.00
Selenium patch event-based	1	0.52	0.50	0.44	1.00
Multiple major c.w.-based	4	0.57	0.51	0.47	1.00
Multiple minor c.w.-based	71	0.57	0.51	0.53	0.01
Multiple patch c.w.-based	4	0.44	0.51	0.46	0.02
Django minor c.w.-based	473	0.39	0.50	0.45	0.54
Django patch c.w.-based	4	0.55	0.50	0.48	1.00
Selenium minor c.w.-based	71	0.57	0.51	0.47	0.10
Selenium patch c.w.-based	406	0.55	0.50	0.54	0.08

In the table we report the number of features after the feature selection step together with ROC-AUC, PR-AUC, F1-score and permutation test p-value measures for hSBM feature space.
