# Supplementary material for: Is it feasible to detect FLOSS version release events from textual messages? A case study on Stack Overflow
Source: PLoS One. 2021 Feb 4;16(2):e0246464. doi: 10.1371/journal.pone.0246464 (PMC7861391; doi:10.1371/journal.pone.0246464)
Supplement: S3 Appendix — (PDF) [file pone.0246464.s003.pdf]

S3 Appendix Synthetic data generator optimization. To ensure maximum similarity between the synthetic and real world datasets, the softmax temperature and top_k parameters of the fine-tuned GPT-2 generator were optimized. These parameters affect the properties of the generator output distribution.

The following two metrics were used to assess similarity between message corpora:

1. Pairwise Jaccard similarity [1] between the posts, which is used in Natural Language Processing in different variations [2];
2. Kullback-Leibler divergence was used for quantitative comparison of the distance distributions, as a well-established method for distributions comparison [3–5].

Since it is not feasible to compute the pairwise distances between all individual messages, we took random samples of 500 posts from the data and repeated this sampling 30 times to get the standard deviations of the result. We also made sure that further increase of the random sample size does not change the optimization outcome.

To our knowledge, there is no unified framework for assessing the synthetic texts quality. However, it is common to consider such measures as Fluency, Novelty, Diversity and Intelligibility of the generated entries [2]. Generating the data for automated processing purposes, we have taken two properties - Novelty and Diversity, which are obtained for the synthetic and the real world datasets.

Based on the equations in [2], Novelty defines distance between the synthetically generated and the real world datasets. Diversity of the dataset can be measured as novelty between two samples of the same dataset. Fluency and Intelligibility are more subtle measures and cannot be directly measured from the defined metrics. Aiming to make the approach simple and universal, we limit the synthetic data assessment to 2 measures.

With this understanding, we have applied the measures to our case:

- Novelty: a sample of the synthetic data is assessed against the sample of the real world data. The metrics value should be as small as possible. Ideally it should be equal to Diversity of the real world dataset.
- Diversity: a data sample is assessed against a different sample within the same dataset. The metrics values should be as similar as possible for the real world and

synthetic data.

The optimization process was performed separately for the event-related and background messages. Then, a single set of parameters was chosen to generate all the messages in order to preserve the consistency of the dataset and avoid the process of differentiating between the positive and negative entries to become trivial.

The optimal configuration was chosen based on the Novelty and Diversity of the event-related and background messages. In the optimization we have treated the quantitative improvements in both measures equally. The consensus set of parameters was expected to have the least Novelty and Diversity differences between the synthetic and the real world data. During the optimization, Novelty of event-related messages changed 1 order less than of the background messages. Diversity changes were comparable. Consequently, choosing the final parameter set we used equal contributions from the two message types for Diversity, and Novelty was chosen with $\tilde{9}0\%$ priority in the background messages.

Also, we observed that 0.1 decrease of the softmax temperature leads to a huge divergence between the synthetic and the real world data properties. Based on Novelty and Diversity optimization and our observations, we have chosen the softmax temperature of 1.0 and the top_k of 400.

References

1. Jaccard coefficient: Jaccard P. Étude comparative de la distribution florale dans une portion des Alpes et des Jura. Bulletin de la Société Vaudoise des Sciences Naturelles. 1901;37.

2. Wang K, Wan X. Sentigan: Generating sentimental texts via mixture adversarial networks. IJCAI International Joint Conference on Artificial Intelligence. 2018;2018-July:4446–4452. doi:10.24963/ijcai.2018/618.

3. Pereira F, Tishby N, Lee L. Distributional clustering of English words. arXiv preprint cmp-lg/9408011. 1994;.

4. McCallum A, Nigam K. Employing EM and Pool-Based Active Learning for Text Classification. Proceedings of the Fifteenth International Conference on Machine Learning. 1998; p. 350–358.

5. Becker M, Osborne M. A two-stage method for active learning of statistical grammars. IJCAI International Joint Conference on Artificial Intelligence. 2005; p. 991–996.
