# Supplementary material for: Is it feasible to detect FLOSS version release events from textual messages? A case study on Stack Overflow
Source: PLoS One. 2021 Feb 4;16(2):e0246464. doi: 10.1371/journal.pone.0246464 (PMC7861391; doi:10.1371/journal.pone.0246464)
Supplement: Attachment — Submitted filename: response_to_reviewers.pdf [file pone.0246464.s004.pdf]

Artur Sokolovsky
Newcastle University
1, Science Square
Newcastle upon Tyne,
NE45TG, UK
+44 07480 317713
artur.sokolovsky@gmail.com

Editors
PLoS ONE
October 17, 2020

Dear Dr Soldani,

We would like to thank you and reviewers for the work you have put into the thorough assessment of our manuscript as well as for the opportunity to improve and resubmit our research. We have made the best use of the comments by running additional experiments, extending and restructuring the manuscript. On the following pages, we are responding to the editor and reviewers.

To make it easier distinguishing comments and answers, in section 2 we color the comments in grey.

1) EXECUTIVE SUMMARY OF CHANGES

- Improved the structure of the manuscript;
- Corrected the typos, language and formatting;
- Added LDA optimization plot (Fig. 13);
- Extended the experiments and analysis by adding an extra feature space (hSBM-based) and a third estimator (CatBoost);
- Extended the Background section by incorporating the suggested studies;
- Changed the title of the study;
- Modified the Abstract.

2) REPLY TO THE COMMENTS

- Reply to the editor's comments

1. Please ensure that your manuscript meets PLOS ONE's style requirements, including those for file naming. The PLOS ONE style templates can be found at

https://journals.plos.org/plosone/s/file?id=wjVg/PLOSOne_formatting_sample_main_body.pdf and

https://journals.plos.org/plosone/s/file?id=ba62/PLOSOne_formatting_sample_title_authors_affiliations.pdf

We have corrected the formatting according to the requirements.

2. Please clarify whether all terms and conditions of the websites, software and datasets used were complied with in the data collection and data sharing processes.

CC BY-SA 4.0 license is applied to Stack Overflow message data.

Libraries.io data is licensed by CC-BY-4.0.

3. We note that Figures in your submission contain copyrighted images. All PLOS content is published under the Creative Commons Attribution License (CC BY 4.0), which means that the manuscript, images, and Supporting Information files will be freely available online, and any third party is permitted to access, download, copy, distribute, and use these materials in any way, even commercially, with proper attribution. For more information, see our copyright guidelines: http://journals.plos.org/plosone/s/licenses-and-copyright.

To our knowledge, we do not use any copyrighted images. Please let us know which figures are copyrighted.

Draw.io was used to create diagrams and it has a statement regarding licensing: (https://desk.draw.io/support/solutions/articles/16000042494-usage-terms-for-diagrams-created-in-diagrams-net). Please let us know if this is not enough.

- ## Reply to reviewer 1

We are very glad that you find our research worth publishing in the journal. We respond to all your comments below:

• The article requires improvement in terms of structure, organisation and flow of information. As it is, the manuscript falls short of providing a clear and easy to follow structure of the study and proposed models/processes, and the reader has to read the article multiple times to obtain a full-picture of the whole reported work and how each process leads to the next. The descriptions seem to go back and forth in places and,

hence, the authors need to make sure that the article provides, with the help of better illustrations, simple description of the various phases of the study, their sequences and associated processes, and how each leads to the next.

Thank you for pointing at this crucial aspect - we realize the importance of the readability of the paper and have done our best to improve it. Concretely: we have modified the structure of the study by restructuring Introduction and Background sections as well as editing the rest of the manuscript. Additionally, we have added extra figures to improve the flow of the paper (Figs. 1, 3).

- *The article in my opinion needs some focus in its aim: initially, and as the title indicates, the focus seems to be on developing models for a successful micro-events detector/estimator. However, later on and once the performance of the estimators was reported to be insignificant, the focus seems to shift predominantly to 'better understand the data' and generating synthetic data to find the detectability threshold. Again, I would like to emphasize the quality and standard of presented work here, however the authors should also declare a clear aim/focus of this article one way or another right from the beginning, reflect that in tile properly and stick to this aim.*

We have changed the title of the paper. Additionally, after adding an extra set of experiments (corrected for multiple comparisons), we have found a number of significant models. Consequently, we rejected the null hypothesis. Finally, we modified the Abstract focusing it more on the result. We hope that these aspects make the manuscript more uniform.

- *The developed synthetic data generator is one of the main contributions of this work. However, I am not sure how such a finely tuned and controlled synthetic dataset is going to help the development of a detector that generalises to a non-synthetic domain without fine-tuning on any real world data.*

We currently fine-tune the generator on the real world data. All the generator parameter tweaking is aimed at replicating the distributions of the real world dataset. This is expected to ensure the generalizability of the synthetic data. Following the proposed procedure one would be capable of generating a synthetic dataset for other real world datasets . Currently, we use two metrics to ensure that the synthetic dataset is as close to the real world data distributions as possible (namely Novelty and Diversity). As the future work, we suggest in the manuscript to look into other metrics for assessing the datasets similarity. This might further improve the generalizability.

• Relating to above, I would have liked to see the authors focusing more on improving the performance of the proposed detector pipeline via, for example, attempting and comparing other topic modelling techniques such as LSA, pLSA and Deep LDA, etc., and adjusting the NLP tools of the sentiment analysis to targeted domain (issues the authors have already discussed as limitations of the study). In this respect, the authors should also try to deviate a bit from their target to limit the number of variables in the feature space (which seems to me solely based on the claim of 'curse of dimensionality', which is known to be dataset and algorithm dependent and has well-established remedies).

In order to ensure that the results are not influenced by the sub-optimal method selection, we have extended the study by including hSBM features as an alternative novel topic modeling approach. Additionally, we have added an extra classifier - CatBoost, a state of the art boosting technique. We did not alter the sentiment analysis tool, but better explained our choice in the manuscript.

• Manuscript contains few typos and I suggest the authors proofread it again and correct (quick example is on p.8 in the Model Performance Section: '… we compute a mean of PR-AUC - event as a positive label and no-event is a positive label.')

We have made the best effort to improve the language as well as spot and correct the typos.

• I have noted that this exact and full manuscript currently appears on: 1) the arXiv archive by Cornell University (at https://arxiv.org/abs/2003.14257), and on researchgate.net (at https://www.researchgate.net/publication/340331989_Detection_of_FLOSS_version_release_events_from_Stack_Overflow_message_data), and would like to advise the authors to check with PLOS ONE publications policy and terms and conditions.

Thank you for the advice. We have never published our research on researchgate.net - they could obtain it directly from arXiv without our knowledge/permission. From what we know, PLOS ONE encourages authors to submit pre-prints.

- **Reply to reviewer 2**

1. The introduction is rather like a report and contains different parts of information. I suggest the authors to improve the flow in the text and connect with logical cohesion the different parts.

Thank you for the comment - we have made the best effort to restructure the Introduction section.

2. Also, it is not mandatory, but most research approaches form more than one RQs. You could reform the structure and add more research questions i.e. evaluating which is the best ML algorithm to perform event detection in SO post.

We appreciate your comment. In order to formally answer the suggested research question we would need to run additional statistical tests. Since we feel that the paper is already complex, we would not add more research questions at this point.

3. Similar to the introduction section also the "Background material and related work" section needs better connection between paragraphs.

We have restructured the Background section. Additionally, we made the best effort to improve the language throughout the manuscript.

4. Additionally, for the related work it could be helpful for readers to add a comparison between different algorithms used to identify events from different online sources such as the Twitter (i.e. https://dl.acm.org/doi/10.1145/1978942.1978975). Identify which is the target source, which is the data analysis method, which option of event detection authors used etc. and compare with your own research.

To address the comment, we have added more relevant studies to the Background section and communicated their differences to the current work. We believe that the definition of the event contributes more to the performance than the estimator choice. Since we reintroduce the notion of micro-events, we feel that our research results cannot be directly compared to the other studies.

5. Additionally for the bibliography part there are many works which use LDA in Stack Overflow posts (e.g., https://link.springer.com/article/10.1007/s10664-012-9231-y or

https://link.springer.com/article/10.1007/s11390-016-1672-0). I would expect to see some discussion for this in the bibliography part.

Thank you for suggesting these works - we included them into the Background section.

6. Moreover there are work which identify from Stack Overflow posts technology releases i.e. http://das.encs.concordia.ca/uploads/abdellatif_msr2020.pdf (fig 3). So, I would expect to see a reference for this as well.

This is a very good point - we have mentioned this study in the Background section.

7. In "Pre-processing" subsection for sentiment analysis calculation please explain why you select to use Vader method and not use a sentiment analysis approach which is constructed for developers' post such as https://link.springer.com/article/10.1007/s10664-017-9546-9

We appreciate the comment - we have explained our choice in the Pre-processing subsection of Materials and methods section.

8. Additionally, in the same subsection you can add a graph which depicts the results of coherence measure used to select the appropriate number of topics (i.e. https://link.springer.com/article/10.1007/s10664-020-09819-6 see Fig. 12)

Thank you for the comment - we have added the LDA model optimization figure (Fig. 13).

9. In the "Analysis pipelines" subsection the analysis for the construction of Logistic Regression and Random Forest is good. However why do you select this ML method? How about an SVM approach?

This is a very good point - to ensure that we are making the best effort in terms of machine learning, we extended the study by running the pipeline with CatBoost estimator. We preferred using decision tree-based techniques since they are robust and directly interpretable. SVMs are harder to interpret in case of larger dimensionalities. Also, in contrast to CatBoost, they are quite sensitive to parameter tuning.

10. I suggest adding a section after "Limitation" section with implications for practitioners. For example, use case scenarios on how someone can use your research approach to find a new FLOSS version.

Thank you for your suggestion - we have added it.

3) CLOSING STATEMENT

We thank you for your consideration of this resubmission. After addressing the reviewer's comments we believe the manuscript is much stronger now and hopefully deserving of acceptance.

Sincerely,

Artur Sokolovsky (corresponding author)
artur.sokolovsky@gmail.com

Thomas Gross, PhD
thomas.gross@ncl.ac.uk

Jaume Bacardit, PhD
jaume.bacardit@ncl.ac.uk
